# Adherence to the dietary approaches to stop hypertension diet reduces the risk of diabetes mellitus: a systematic review and dose-response meta-analysis

**DOI:** 10.1007/s12020-024-03882-5

**Published:** 2024-05-30

**Authors:** Xiyan Quan, Xiaoming Shen, Chun Li, Yayuan Li, Tiangang Li, Baifan Chen

**Affiliations:** Department of Endocrinology, Pinghu First People’s Hospital, Pinghu, 314200 Zhejiang Republic of China

**Keywords:** Diabetes mellitus, Dietary Approaches to Stop Hypertension, DASH diet, Systematic review, Meta-analysis, Epidemiology

## Abstract

**Background:**

Despite several epidemiological studies reporting a significant association between adherence to the Dietary Approaches to Stop Hypertension (DASH) diet and the risk of diabetes mellitus, the results remain controversial. In this systematic review and meta-analysis, we aimed to summarize the existing evidence from published observational studies and evaluate the dose-response relationship between adherence to the DASH diet and diabetes mellitus risk.

**Methods:**

We performed a systematic search for relevant articles published up to September 2023 using electronic databases of PubMed, Embase, Scopus, and China National Knowledge Infrastructure (CNKI). A random-effects model was applied to calculate the combined relative risks (RR) with 95% confidence intervals (CIs) for the highest compared to the lowest categories of DASH score in relation to diabetes mellitus risk. Heterogeneity among the included studies was assessed using the Cochran’s *Q* test and I-squared (*I*^*2*^) statistic. Literature search, study selection, data extraction, and quality assessment were performed by two independent reviewers.

**Results:**

Fifteen studies involving 557,475 participants and 57,064 diabetes mellitus cases were eligible for our analyses. Pooled analyses from included studies showed that high adherence to the DASH diet was significantly associated with a reduced risk of diabetes mellitus (RR: 0.82; 95% CI: 0.76–0.90, *P* < 0.001). Moreover, the dose-response meta-analysis revealed a linear trend between adherence to the DASH diet and diabetes mellitus (RR:0.99; 95%CI: 0.97–1.02, *P*_*dose-response*_ = 0.546, *P*_*nonlinearity*_ = 0.701). Subgroup analyses further revealed a significant inverse association between adherence to the DASH diet and diabetes mellitus risk in case-control studies (RR: 0.65; 95%CI: 0.29–1.43, *P* < 0.001), with a marginal inverse association in cohort studies (RR:0.83; 95%CI: 0.76–0.91, *P* < 0.001). Additionally, we conducted analyses separately by comparison and found a significant inverse association between DASH diet and diabetes mellitus risk in T3 vs T1 comparison studies (RR = 0.74; 95%CI: 0.64–0.86, *P* = 0.012).

**Conclusion:**

The findings of this study demonstrate a protective association between adherence to the DASH diet and risk of diabetes mellitus. However, further prospective cohort studies and randomized controlled trials are needed to validate these findings.

## Introduction

Type 2 diabetes mellitus (DM) represents a major global health problem, which has become a severe, chronic, non-communicable disease after cardio-cerebrovascular diseases [1]. According to the statistics reported by the International Diabetes Federation (IDF) in 2021, approximately 537 million adults aged 20–79 years worldwide were affected by Type 2 DM, and this number is projected to 784 million by 2045 [2]. Additionally, the latest release global DM map reveals that China alone had 140.9 million people with DM in 2021, accounting for a quarter of the total global DM patients [3]. It is well-known that lifestyle risk factors, such as obesity, cigarette smoking, sedentary behavior, and unhealthy eating habits, play an important role in the prevention of DM [4]. Therefore, making appropriate food choices and adopting a healthy diet are essential strategies to prevent or delay the onset of DM [5].

In the past several decades, a growing body of evidence supports the potential role of diet in the prevention of DM [6–8]. Many epidemiological studies have specifically reported the links between the intakes of specific nutrients or foods, such as vegetables, fruit, and whole grains, and the risk of DM [9, 10]. However, due to the complex interactions between dietary components and the potential synergistic effects of nutrients and foods consumed together, previous studies have revealed only a limited influence of diet on DM risk [11]. Given that, dietary pattern analysis, which takes into account the complexity of whole diet, has been widely applied in nutritional epidemiology [12]. Consistently, the results of dietary pattern analysis could be more easily translated into national dietary guidelines [7].

For instance, the Dietary Approaches to Stop Hypertension (DASH) diet, originally developed for the management of high blood pressure, has been proposed to potentially lower blood glucose levels [13]. The DASH diet, a well-established healthy dietary pattern, emphasizes high consumption of fruits, vegetables, whole grains, nuts, and legumes, moderate consumption of low-fat dairy products, as well as low consumption of sodium, sweetened beverages, and red and processed meats [14]. Notably, the DASH diet has been recommended as one of three healthy dietary patterns in the 2020–2025 United States Dietary Guidelines for the public [15].

To date, many epidemiological studies have found that adherence to the DASH diet is significantly associated with several non-communicable diseases, such as hypertension, cardiovascular disease, chronic kidney disease, and certain types of cancer [16–18]. Notably, less is known about the influence of the DASH diet on the risk of DM. In 2009, Liese et al. published the first study reporting the association between adherence to the DASH diet and type 2 DM in the multiethnic Insulin Resistance Atherosclerosis Study [8]. Since then, there have been considerable attentions in medical research on the relationship between greater adherence to the DASH diet and risk of DM [7, 8, 19, 20]. However, the results are not entirely consistent. Although some epidemiological studies have shown the protective role of the DASH diet in the development of DM [7, 8, 19, 21], others have found no association [20, 22]. Moreover, an earlier meta-analysis of 20 randomized controlled trials (RCTs) on the DASH diet and its impact on type 2 DM risk demonstrated that the DASH diet could significantly reduce fasting insulin concentration [23]. Furthermore, to the best of our current knowledge, there is no published systematic review and dose-response meta-analysis evaluating the effect of adherence to the DASH diet on DM risk. Therefore, we performed a systematic review and dose-response meta-analysis of observational studies published from inception until September 2023 to assess the potential impact of adherence to the DASH diet on DM risk.

## Materials and methods

We followed the Meta-Analysis of Observational Studies in Epidemiology (MOOSE) and Preferred Reporting Items for Systematic Reviews and Meta-Analysis (PRISMA) guidelines for reporting this study [24]. A protocol has been registered in the International Prospective Register of Systematic Reviews (CRD42023465848).

### Search strategy

An electronic literature search *via* four databases, including PubMed, Embase, Scopus, and CNKI was performed to find related publications written in the English or Chinese languages published from their dates of inception up to September 2023, with the following terms: {[“diabetes” (all fields) OR “diabetes mellitus” (all fields)] AND [“DASH score” (all fields) OR “DASH diet” (all fields) OR “DASH” (all fields) OR “Dietary Approaches To Stop Hypertension” (all fields) OR “Dietary Approaches To Stop Hypertension” (MeSH)]}. Moreover, we also found potentially related articles by manually searching the reference lists of retrieved articles and previously published reviews. The current search strategy was carried out by two independent reviewers (B.-F.C and X.-Y.Q).

### Study selection

Two reviewers (B.-F.C and X.-Y.Q) independently screened the titles and abstracts of potential articles retrieved during the initial search, and ascertained studies exploring the relationship between adherence to the DASH diet and DM risk. After all reviewers agreed, the full-text versions of articles were reviewed according to the inclusion and exclusion criteria for the present systematic review and meta-analysis. The following studies were eligible for our analyses: (1) observational studies (e.g., case-control, cohort, or cross-sectional studies) performed in participants aged ≥18 years; (2) reported the data on the association between adherence to the DASH diet and DM risk; (3) provided the multivariable adjusted RRs, HRs, ORs with their corresponding 95%CIs of DM for the highest versus the lowest categories of DASH diet score (or sufficient data to calculate them); (4) If the data in the retrieved article lacked sufficient detail, the corresponding author of the original study is contacted via email; (5) DM diagnoses were confirmed by clinical interviews or self-report on a previous physician- made diagnosis of DM. Also, studies were excluded if they met one of the following criteria: (1) non-observational studies, e.g., letters, editorials, conference abstracts, reviews, or case reports; (2) studies written in non-English or non-Chinese; (3) irrelevant articles; (4) HRs, RRs or ORs with 95%CIs were not reported in the study. Any disagreements were settled by discussion or in consultation with the third reviewer (X.-M.S). The study population, exposure, comparison, outcome, and study design (PEICO) are shown in Table S1.

### Data extraction

Two independent reviewers (B.-F.C and X.-Y.Q) extracted the following information from the selected studies: the first author’s last name, publication year, location, study design, total number of participants, number of DM cases, mean age/age range for cases and participants, dietary assessment method, the factors that were adjusted or matched for in analyses, and reported risk estimates (HRs/ ORs/RRs) and their corresponding 95%CIs for highest versus lowest categories of DASH diet score.

### Quality assessment

The Newcastle-Ottawa Quality Scale(NOS) was applied by two reviewers to independently assess the quality of included studies in this meta-analysis [25]. According to this scale, each study could be assigned a maximum score of 9 points for three main domains: selection(range 0–4 points), comparability (range 0–2 points), and assessment of outcomes (range 0–3 points). Studies scoring 7–9 points, 4–6 points, and 0–3 points, were identified as being high, medium, and low quality, respectively [26]. Disagreements were resolved by discussion to reach a consensus.

### Statistical analysis

In this meta-analysis, we used RRs and 95%CIs as the effect size for the main analyses. Besides, ORs were converted into RRs using the following formula: RR = OR/[(1 − P_0_) + (P_0_*OR)], in which P_0_ shows the incidence of DM in the non-exposed group [27]. Subsequently, the RRs and corresponding 95% CIs for comparing incident DM between the highest and the lowest categories of DASH diet scores, were used to calculate log-transformed RRs with their corresponding standard errors (SEs). The Cochran’s Q test and I^2^ statistic were utilized to evaluate the potential sources of between-study heterogeneity. *P*-values of Cochran’s Q test >0.10 or I^2^ > 50% were considered to indicate high heterogeneity among the included studies, and then a random-effects model(DerSimonnian and Laird method) was used to pool the RRs. Otherwise, a fixed-effects model was used to calculate the pooled RRs [28]. If high heterogeneity was present, sensitivity and subgroup analyses were used to explore potential sources of heterogeneity. Subgroup analyses were performed based on comparison (Q5 vs Q1/Q4 vs. Q1/T3 vs. T1), mean age (>50 y/<50 y), country (Western countries/Asian countries), and study design (cohort/case-control studies). If more than 10 studies were available, publication bias was assessed through the visual inspection of the funnel plots and quantified by both Begg’s and Egger’s tests [29]. Sensitivity analyses were performed to explore the extent to which the pooled RRs might be affected by a single study or a group of studies. More importantly, we also performed a dose-response meta-analysis to estimate the RRs for each 1-score increase in DASH diet adherence. A two-stage GLST model based on generalized least squares was used to examine potential linear or non-linear dose-response relationship between adherence to the DASH diet and DM risk. All data analyses were carried out using STATA, version 11.2 (Stata Corp, College Station, TX). A 2-sided *P* value < 0.05 was considered statistically significant.

## Results

### Search results

**Fig. 1:**
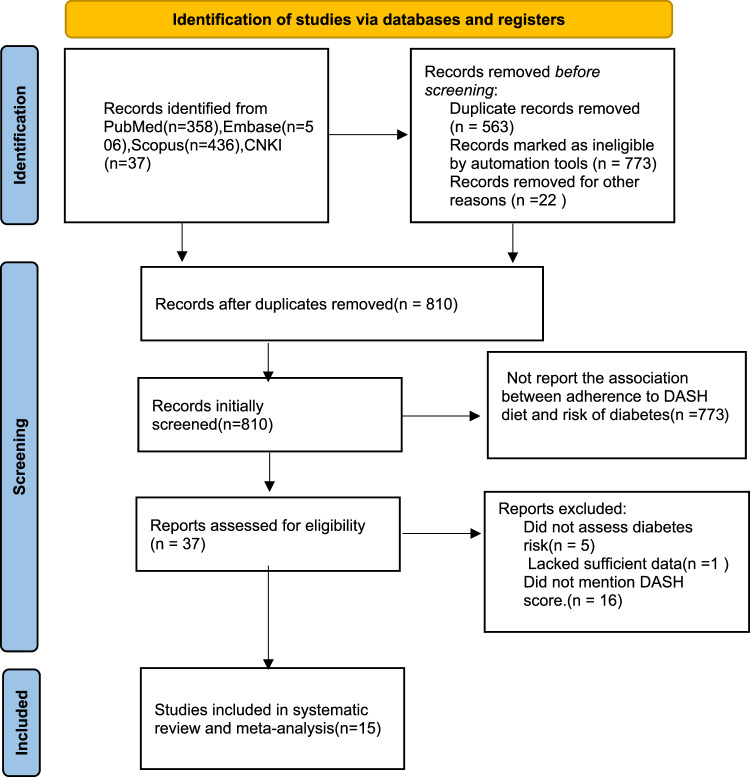
Flow chart of the process of study selection

In the initial search, we retrieved 1373 articles through four database search and the reference lists of included studies and previously published reviews. After removing 563 duplicates, 810 articles were left for further screening. Consistently, reading titles and abstracts of these articles led to the exclusion of 773 articles because they didn’t report the relationship between adherence to DASH diet and risk of DM. Accordingly, the remaining thirty-seven full-text articles were examined for eligibility, and 22 were excluded for the following reasons: 5 did not evaluated DM risk; 1 lacked sufficient data and the corresponding author of this study could not be contacted; 16 did not mention DASH diet score. Finally, fifteen studies(13 cohort and 2 case-control studies) with 557,475 participants and 57,064 cases of DM were included for this systematic review and meta-analysis [6–8, 19, 21, 30–33, 22, 20, 34–37]. Figure 1. indicated the flow chart of article selection process.

### Study characteristics

Characteristics of each eligible study are shown in Table 1. Of these included studies, thirteen were cohort studies [7, 8, 19, 21, 30–33, 22, 20, 34–36], and two were case-control studies [6, 37]. The publication dates of these studies mentioned above varied between 2009 and 2023. Age of study participants ranged from 18 to 84 years. Sample size ranged from 334 to 166500. Eight of the included studies were performed in the United States [7, 8, 19, 21, 30, 22, 32, 33], three in Iran [6, 20, 34], one in Taiwan China [35], one in Singapore [31], one in Brazil [36], and one study in Europe [37]. Fourteen of included studies used food frequency questionnaires(FFQs) to collect dietary data [7, 8, 19, 21, 30–33, 22, 20, 34–37], and remaining one study used 24-h dietary recall [6]. In addition, all included articles used methods designed by Fung et al. (7 food groups and sodium) [7, 30–33, 22, 20, 34, 36], Dixon et al. (7 food groups, saturated fat and alcohol) [6], Günther et al. (8 food groups) [16], Sacks et al. (7 food groups and sodium) [19, 21, 35, 37] to extract DASH diet. Finally, based on the NOS scores, all of the included studies were considered to be of high-quality studies [6–8, 19, 21, 30–33, 22, 20, 34–37].

**Table 1:** Characteristics of included studies on the association between DASH diet and risk of DM

Author publication year	Location	Study design	Total number of participants	Age	Exposure assessment	Follow up year	Diabetes assessment method	Adjustment or matched for analyses	RR(95%CI) for highest versus lowest category
Izadi et al. [6]	Iran	Case-control	200 cases 263 controls	22–44 y	24 h dietary recalls	–	Fasting glucose >95 mg/dL or 1 h postprandial glucose >140 mg/dL for the first time in the pregnancy.	Age, energy, number of children, and socioeconomic status	OR:0.29 (0.17–0.48)
Jacobs et al. [7]	USA	Cohort	89,185 (11,217 cases)	45–75 y	FFQ	7 years	Repeated outpatient visits for diabetes, clinical information, pharmacy records, and hospital discharge diagnoses	Age, adjusted for physical activity, smoking, years of education, total energy intake and BMI	Male: 0.79 (0.73–0.87) Female:0.77 (0.70–0.84)
Liese et al. [8]	USA	Cohort	862 (148 cases)	40–69 y	FFQ	5 years	Diabetes on their oral glucose tolerance test or who were taking hypoglycemic medication	Age, sex, race/ethnicity/ clinic, glucose tolerance status, family history of diabetes, education, smoking status, energy intake, and energy expenditure.	OR:0.64 (0.37–1.13)
Tobias et al. [19]	USA	Cohort	4413 (491cases)	24–44 y	FFQ	14 years	1 or more classic symptoms (i.e., excessive thirst, polyuria, unintentional weight loss, and hunger) and a fasting plasma glucose concentration of 140 mg/dL or more (to convert glucose levels to millimoles per liter, multiply by 0.0555) or a random plasma glucose level of 200 mg/dL or more; no symptoms but 2 or more elevated plasma glucose concentrations on more than 1 occasion (fasting level,≥140 mg/dL; random level, ≥200 mg/dL; 2 h oral glucose tolerance test finding, ≥200 mg/dL); or use of hypoglycemic medication (insulin or an oral hypoglycemic agent). Diagnostic criteria were changed in June 1998 to adopt a new diagnostic threshold for a fasting plasma glucose level of more than 126 mg/dL.	Age, total energy intake, parity, age at first birth, race/ethnicity, parental history of T2DM, oral contraceptive use, menopausal status, smoking status, total physical activity, alcohol intake, BMI	0.68 (0.49–0.94)
Tobias et al. [21]	USA	Cohort	15254 (872cases)	24–44 y	FFQ	10 years	The National Diabetes Data Group criteria	Age, total energy intake, gravidity, smoking status, physical activity, sedentary time, parental history of type 2 diabetes, alcohol and prepregnancy BMI	0.66 (0.53–0.82)
Glenn et al. [30]	USA	Cohort	145299 (13943cases)	50–79 y	FFQ	28 years	Physician-diagnosed diabetes treated with oral medication or insulin	Age, region, smoking, study arm, self-identified race, ethnicity, education, marital status, hysterectomy history, physical activity, alcohol intake, energy intake, hypertension status, family history of diabetes, HT use, cholesterol-lowering medication use, and BMI.	0.78 (0.72–0.83)
Chen et al. [31]	Singapore	Cohort	45411 (5207cases)	45–74 y	FFQ	17 years	Physician’s diagnosis of diabetes	Age at baseline interview, sex, dialect group, year of baseline interview, energy intake, body mass index, physical activity, education, smoking, self-reported history of physician-diagnosed hypertension, coffee consumption, alcohol consumption.	0.71 (0.65–0.79)
de Koning et al. [32]	USA	Cohort	41615 (2795 cases)	≥18 y	FFQ	20 years	The National Diabetes Data Group (cases prior to 1998) and the American Diabetes Association (cases after 1998)	Smoking, physical activity, coffee intake, family history of type 2 diabetes, BMI, and total energy.	0.75 (0.65–0.85)
Jacobs et al. [33]	USA	Cohort	166550 (9200cases)	45–75 y	FFQ	13 years	Self-reported T2D, Medicare claims, a 2007 linkage with diabetes care registries of the two major health insurance plans in Hawaii, and hospital discharge diagnosis data in California	Age, physical activity, smoking status, education, total energy intake and BMI	Male: 0.81 (0.75–0.88) Female: 0.80 (0.73–0.86)
Otto et al. [22]	USA	Cohort	5160(588cases)	45–84 y	FFQ	5 years	New fasting glucose ≥126 mg/dL or the new use of insulin or oral hypoglycemic medications	Age, sex, race/ethnicity, education, field center, smoking status, energy, alcohol use, physical activity, dietary supplement use, BMI and baseline waist circumference	1.02 (0.79–1.30)
Mirmiran et al. [20]	Iran	Cohort	334(133cases)	≥ 21 y	FFQ	9 years	FSG ≥ 126 mg/dL or 2 h-SG ≥ 200 mg/dL, or using glucose-lowering medications.	Age, sex, T2DM-risk score, weight changes, smoking, and physical activity	OR:1.03 (0.96–1.11)
Esfandiar et al. [34]	Iran	Cohort	6112(549cases)	≥18 y	FFQ	6.6 years	FBG concentrations ≥126 mg/dL or 2-hour plasma glucose concentrations ≥200 mg/dL, or treatment with antidiabetic medications.	Age, sex, diabetes risk score, physical activity, smoking, dietary fiber, and total energy intake.	1.13 (0.88–1.46)
Lin et al. [35]	China(Taiwan)	Cohort	4705(995cases)	≥20 y	FFQ	15 years	Two outpatient visits or one hospital admission with a T2DM diagnosis; both fasting plasma glucose ≥126 mg/dl and hemoglobin A1c ≥ 6.5%	Age, sex, current smoking status, alcohol drinking, BMI, exercise, sedentary time and waist circumference, systolic blood pressure, family history of diabetes, marital status, an education level, and average monthly income	0.97 (0.80–1.17)
Drehmer et al. [36]	Brazil	Cohort	9835(1044cases)	35–74 y	FFQ	3 years	Fasting glucose ≥126 mg/dL, 2-h postload glucose ≥200 mg/dL, or HbA1c ≥ 6.5%.	Age, sex, race, education, family income, occupational status, study center, menopausal status, family history of diabetes, BMI, physical activity, current and previous smoking status, alcohol and calorie intake.	OR:1.07 (0.84–1.36)
InterAct et al. [37]	Europe	Case-control	9682 cases 12595 controls	25–79 y	FFQ	–	A review of the existing EPIC datasets at each center using multiple sources of evidence including self-report, linkage to primarycare registers, secondary-care registers, medication use (drug registers), hospital admissions, and mortality data. Rather than self-report, cases in Denmark and Sweden were identified via local and national diabetes and pharmaceutical registers	Age, study center, sex, physical activity, smoking status, education, total energy intake, BMI, and waist circumference	0.95 (0.84–1.07)

*BMI* body mass index, *FFQ* food frequency questionnaire, *USA* United States

### Adherence to the DASH diet and DM

**Fig. 2:**
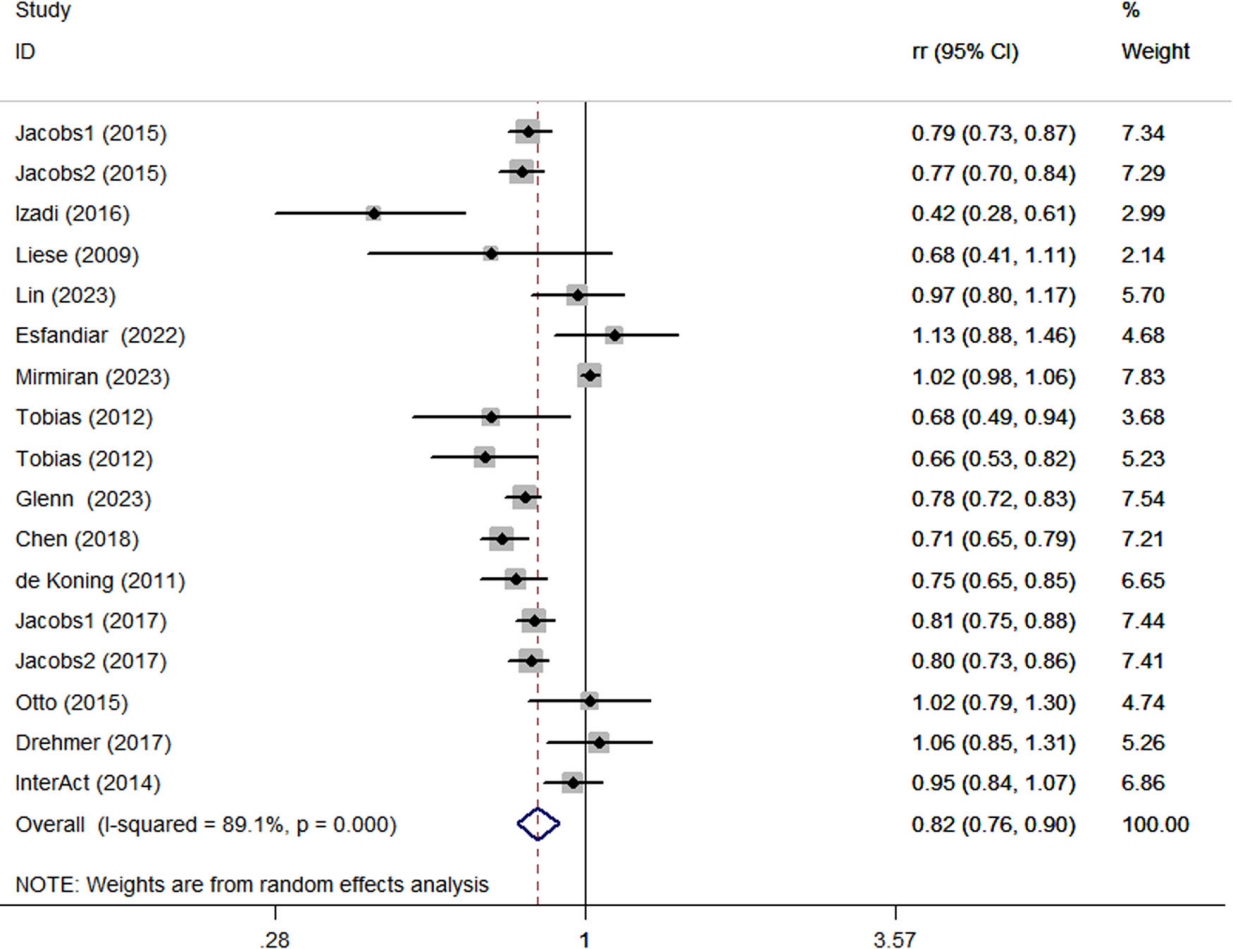
Forest plot of the association between adherence to the DASH diet and risk of DM

Combining 17 effect sizes fSacksrom fifteen articles (557,475 participants and 57,064 cases of DM) were included to evaluate the relationship between adherence to the DASH diet and risk of DM in this study. Figure 2 showed the evidence of a reduced risk of DM in the highest compared with lowest categories of DASH diet (RR:0.82; 95% CI: 0.76, 0.90, *P* < 0.0001). The high heterogeneity was found among the included studies (*P* < 0.0001; I^2^ = 89.1%) and hence the effect was assessed using a random-effects model.

### Dose-response analysis

**Fig. 3:**
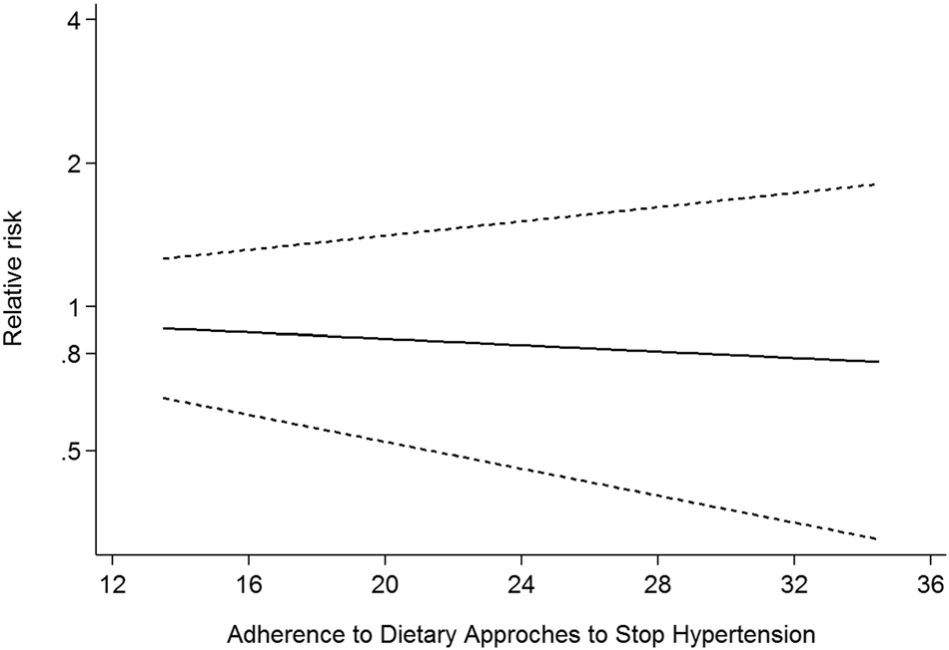
Dose-response association between adherence to the DASH diet and risk of DM in the analysis of twelve studies

**Fig. 4:**
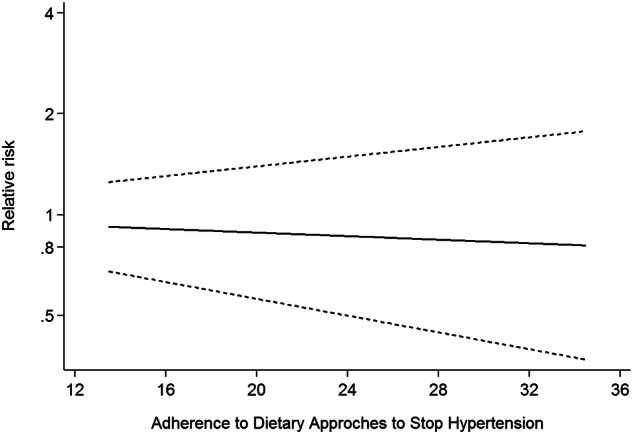
Dose-response association between adherence to the DASH diet and risk of DM in the analysis of cohort studies

Twelve studies [6, 8, 19–21, 31, 32, 34–37] involving 11 cohort studies with DASH diet scores, were included in this dose-response analysis for DM risk. The dose-response meta-analysis showed a linear trend association between adherence to the DASH diet and DM risk (RR:0.99; 95%CI: 0.97–1.02, *P*_*dose-response*_ = 0.546, *P*_*nonlinearity*_ = 0.701) (Fig.3). There was also a linear trend association between DASH diet and DM risk in the analysis of cohort studies (*P*_*nonlinearity*_ = 0.482, *P*_*dose-response*_ = 0.599) (Fig.4).

### Subgroup analyses

**Fig. 5:**
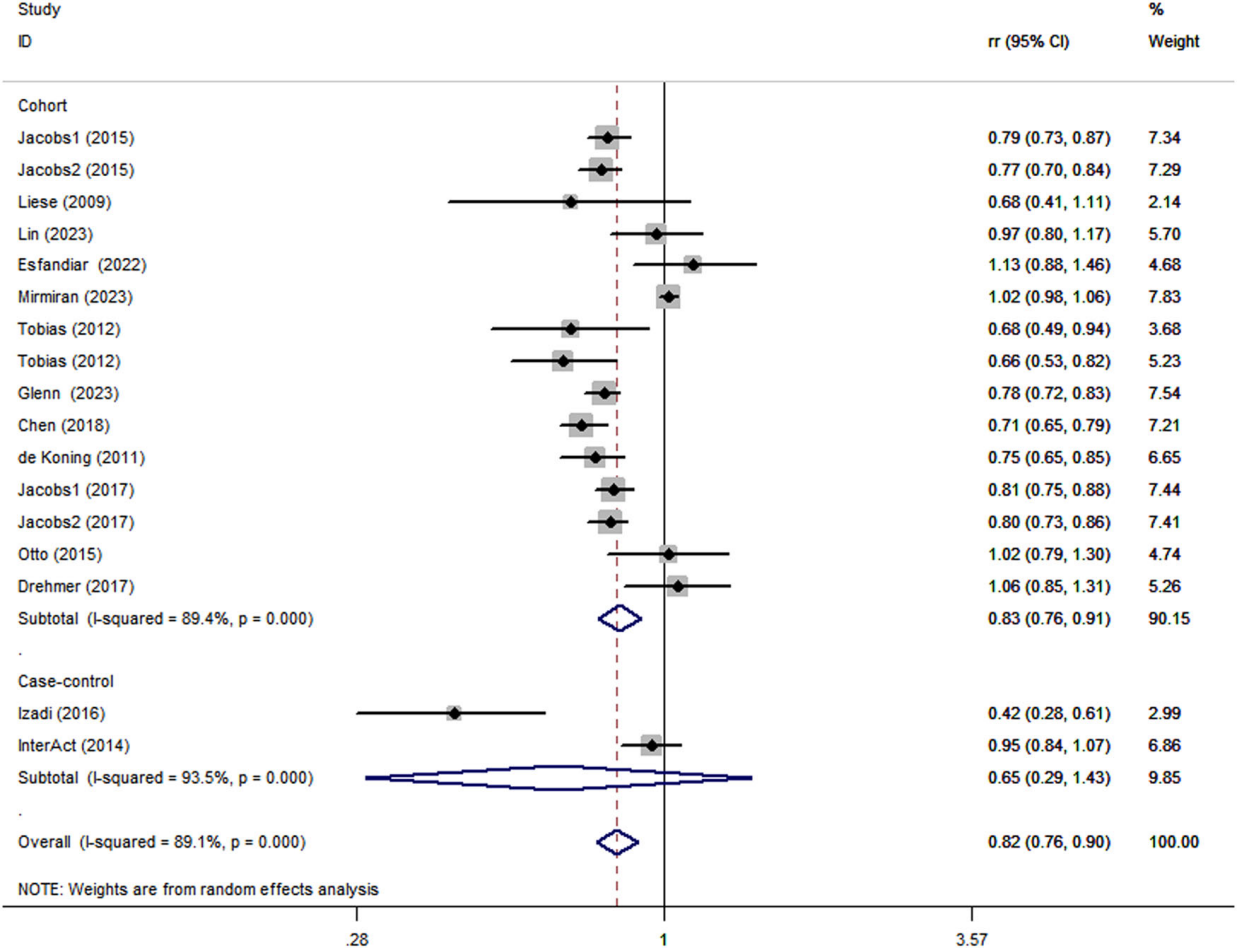
Subgroup analyses of DASH diet and risk of DM according to study design

**Fig. 6:**
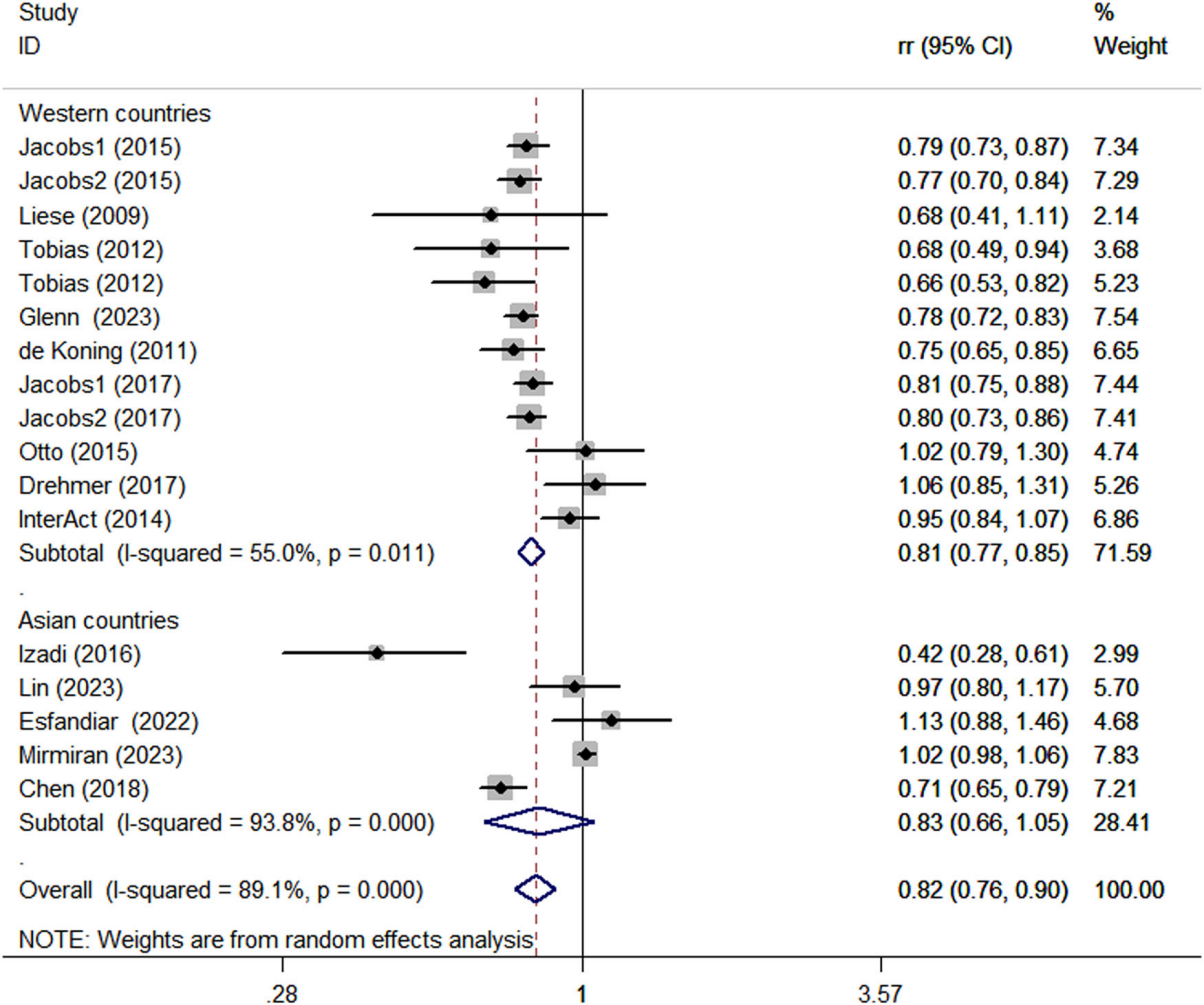
Subgroup analyses of DASH diet and risk of DM according to country

**Fig. 7:**
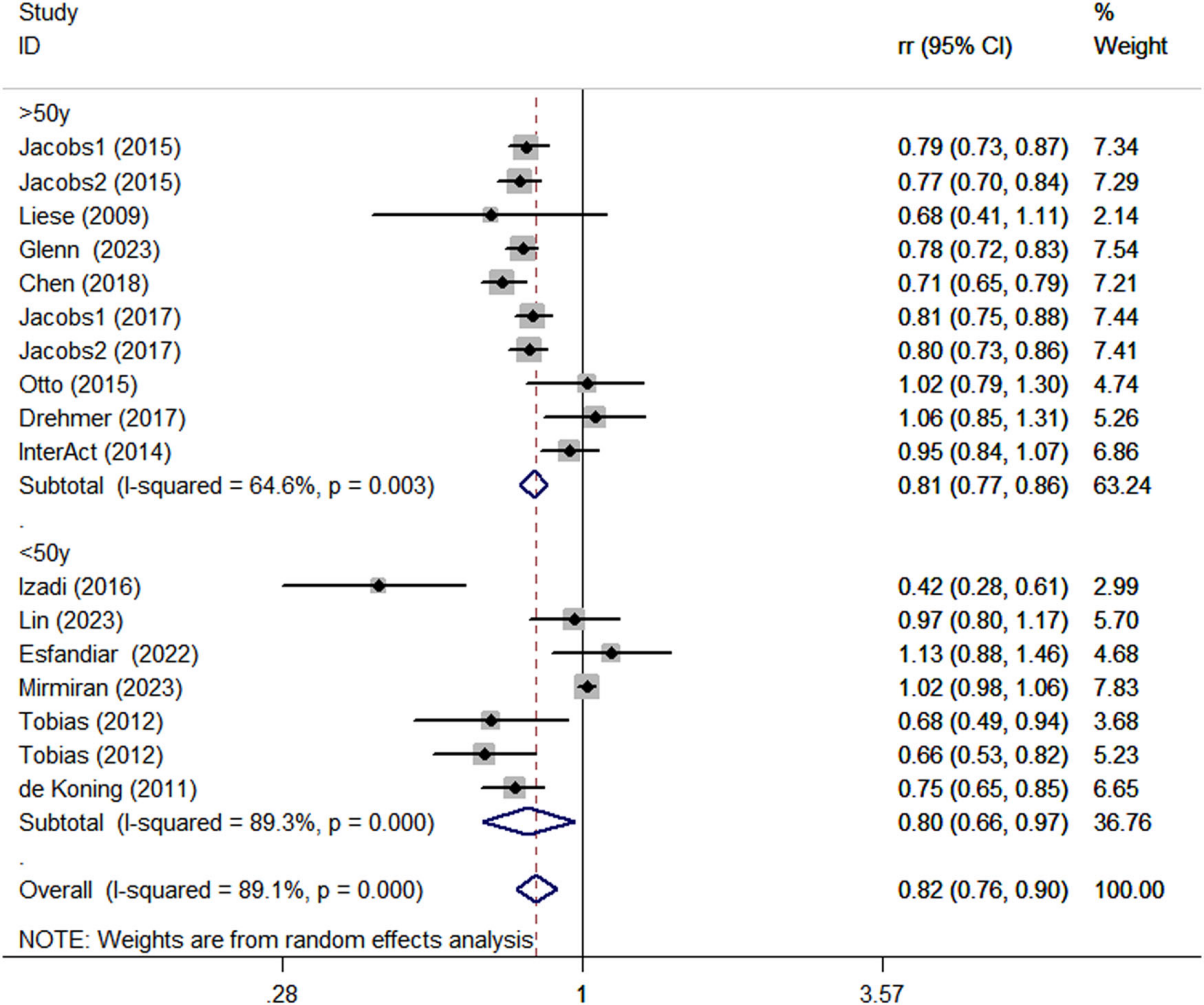
Subgroup analyses of DASH diet and risk of DM according to age

**Fig. 8:**
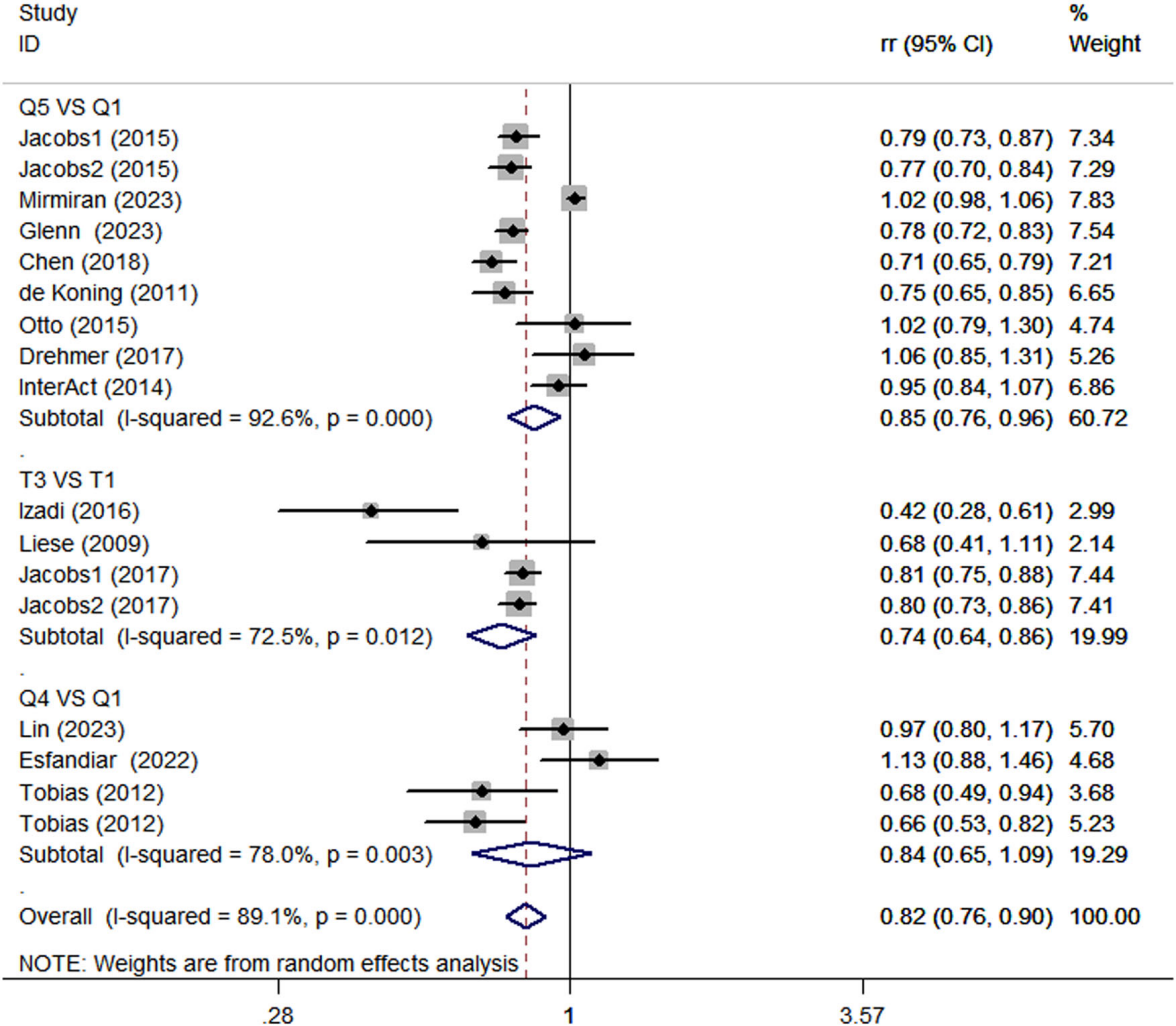
Subgroup analyses of DASH diet and risk of DM according to comparison

Considering the high heterogeneity of the present study (*P* < 0.0001; I^2^ = 89.1%), subgroup analyses were performed to further explore the potential sources of heterogeneity among included studies (Table 2). Subgroup analyses was stratified basing on study design (cohort vs. case-control studies), country (Western vs. Asian countries), age (>50 y vs. <50 y), and comparison (Q5 vs.Q1/Q4 vs.Q1/T3 vs.T1). When we conducted analyses separately by study design (Fig. 5), we found a significant inverse association between adherence to the DASH diet and DM risk in case-control studies (RR = 0.65; 95% CI: 0.29–1.43, *P* < 0.001). In the cohort studies, there was a marginally significant association between the DASH diet and risk of DM (RR = 0.83; 95% CI: 0.76–0.91, *P* < 0.001). The stratified association between the DASH diet and risk of DM according to country (based on the random-effects model) is provided in Fig. 6. There was significant heterogeneity in Asian countries, where a decreased risk of DM was shown (RR = 0.83; 95% CI: 0.66–1.05, *P* < 0.001). Similarly, in Western countries, the risk is similarly reduced (RR = 0.81; 95% CI: 0.77–0.85, *P* = 0.011). When we conducted analyses separately by age (Fig. 7), we found a significant inverse association between the DASH diet and DM risk in all age groups (<50 y: RR = 0.80; 95% CI: 0.66–0.97, *P* < 0.001 and >50 y: RR = 0.81; 95% CI: 0.77–0.86, *P* = 0.003). Similarly, we also performed stratified analysis based on comparison in Fig. 8. Among comparison studies, there was more heterogeneity in T3 vs T1 (*P* = 0.012, *I*^2^ = 72.5%), and a significantly decreased risk of DM was shown (RR = 0.74; 95%CI: 0.64, 0.86; *P* = 0.012). In Q5 vs Q1 and Q4 vs Q1, a marginally significant association between DASH diet and risk of DM was shown.

**Table 2:** Subgroup analyses for the association between DASH diet and risk of DM

Study characteristic	Category	No. of studies	I^2^	RR (95%CI)	*P*
Comparison	Q5 vs Q1	8	92.6%	0.85 (0.76, 0.96)	<0.0001
	Q4 vs Q1	4	78.0%	0.84 (0.65, 1.09)	0.003
	T3 vs T1	3	72.5%	0.74 (0.64,0.86)	0.012
Age	>50 y	8	64.6%	0.81 (0.77, 0.86)	0.003
	<50 y	7	89.3%	0.80 (0.66, 0.97)	<0.0001
Country	Western countries	10	55.0%	0.81 (0.77, 0.85)	0.011
	Asian countries	5	93.8%	0.83 (0.66, 1.05)	<0.0001
Study design	Case-control	2	93.5%	0.65 (0.29, 1.43)	<0.0001
	Cohort	13	89.4%	0.83 (0.76, 0.91)	<0.0001
Type	T2D	12	89.5%	0.84 (0.77, 0.92)	<0.0001
	Other types	3	89.8%	0.68 (0.42, 1.09)	<0.0001

*DASH* dietary approaches to stop hypertension, *RR* relative risk, *CI* confidence interval

### Publication bias

The funnel plots showed little evidence of asymmetry (Supplementary Fig. 1), and there is no evidence of publication bias (highest versus lowest categories of DASH diet score: Egger’s test, *P* = 0.088; Begg’s test, *P* = 0.711).

### Quality assessment

The quality of included studies was shown in Appendix 1. All included studies received an NOS score ≥7, and they were considered to be of high-quality studies [6–8, 19, 30–33, 22, 20, 34–37].

### Sensitivity analyses

Based on the results of sensitivity analysis (Supplementary Fig. 2), a cohort study performed by Mirmiran et al. [10] was outside the limit and might be the source of heterogeneity. When this study was excluded in the repeat analysis (Supplementary Fig. 3), the results showed a slight reduction in the pooled RRs of the association between adherence to the DASH diet and DM risk (RR = 0.81; 95%CI: 0.76–0.86, *P* < 0.001). Meanwhile, heterogeneity of included studies has decreased from 89.1% to 71.0%.

## Discussion

To our current knowledge, this is the first systematic review and meta-analysis to quantitatively summarize the published evidence on the association between greater adherence to the DASH diet and the risk of developing DM. Our analysis includes data from fifteen studies, involving 57,064 diabetes cases and 557,475 participants. The results demonstrated that high adherence to the DASH diet was associated with an 18% reduction in the risk of DM. Furthermore, the dose-response analysis showed a linear trend between DASH diet and DM risk. Sensitivity analysis also showed that excluding a certain study did not significantly alter the pooled effect of DASH diet adherence on DM risk. Overall, our findings add to the evidence of an inverse association between adherence to the DASH diet and DM risk and support the adoption of DASH diet adherence as a primary prevention strategy for DM.

Over the past four decades, the global epidemic of DM has continued to escalate [38]. As of 2021, an estimated 537 million adults worldwide are living with diabetes, with 80% of them residing in low- and middle-income countries [3]. This continued upward trend underscores the urgency of preventive measures. Among the various risk factors for DM, dietary factors, especially overall dietary patterns have garnered considerable attention [5]. Researchers have identified the whole dietary patterns using either a priori or a posteriori methods in nutritional epidemiology studies [12]. For example, the DASH diet, as a priori dietary pattern, emphasizes high consumption of fruits, vegetables, whole grains, nuts, and legumes, moderate consumption of low-fat dairy products, as well as low consumption of sodium, sweetened beverages, and red and processed meats [14]. Despite numerous epidemiological studies have investigated the relationship between adherence to the DASH diet and DM risk [20, 22, 33–37], the findings remain inconsistent. For instance, the Iran cohort study by Esfandiar and colleagues reported a 13% increased risk of DM associated with adherence to the DASH diet (RR = 1.13, 95%CI: 0.88–1.46) [34]. Conversely, the Brazilian Longitudinal Study of Adult Health (ELSA-Brasil) found that greater adherence to the DASH diet was not significantly related to the risk of newly diagnosed DM (OR:1.07; 95%CI: 0.84–1.36) [36]. The observed inconsistencies in published studies might be attributed to sociodemographic disparities among study populations and differences in the adjustment for potential confounders. For instance, in a previous systematic literature review and meta-analysis, Jannasch et al found that adherence to the DASH diet may change over time due to shifts in socioeconomic factors including poverty, globalization, and increased access to high-calorie foods [39]. Among the 15 studies included, most adjusted for critical confounding variables, such as age, BMI, physical activity, smoking status, and energy intake. Besides, some studies also adjusted for family history of DM and sex [39]. In our study, nine of the included studies were conducted in Western countries, while six studies were conducted in Asian countries and elsewhere. It is widely recognized that there exist significant differences between Eastern and Western diets [40]. Furthermore, to date, the relationship between adherence to the DASH diet and risk of DM has not yet been studied in a systematic review and dose-response meta-analysis. In this context, identifying the association between adherence to the DASH diet and DM risk through a systematic review and meta-analysis would seem to hold significant value.

Our analyses revealed a significant inverse association between adherence to the DASH diet and DM risk. Consitent with our results, several prior studies have reported an inverse association between “healthy” dietary patterns, which share some similar components with the DASH diet, and the risk of DM [41]. Consistently, a previous meta-analysis of 10 prospective cohort studies, involving 404,528 participants, found that high adherence to “healthy” dietary patterns significantly reduced the risk of type 2 DM (RR:0.86; 95%CI:0.82–0.90) [42]. However, contrary to our results, another studies did not find a significant association between adherence to the DASH diet and DM risk [35, 36]. Although evidence associating DASH diet to DM remains somewhat inconsistent, some possible mechanisms have been reported to explain the observed inverse association. First, the DASH diet emphasizes increased consumption of fruits, vegetables, whole grains, and legumes, all of which are rich sources of dietary fiber. Increasing evidence has suggested that higher intake of dietary fiber was associated with a reduced risk of type 2 DM [43]. Moreover, Lattimer and Haub’s findings underscored that high intake of dietary fiber, particularly soluble fiber, could delay gastric emptying and limit carbohydrate absorption, reducing in lower postprandibular blood glucose and insulin levels [44]. Second, vegetables and whole grains are the most important foods in the DASH diet, both of which have a low glycaemic index and load. A recent systematic review and updated meta-analyses of prospective cohort studies showed a robust association between diets high in glycemic index and glycemic load and reduced risk of type 2 DM [45]. Third, whole grains consumption has been linked to a decrease the risk of overweight and obesity [46], both of which are recognized risk factors for DM [47]. Fourth, numerous studies have suggested that antioxidants, including vitamin C and carotenoids abundant in vegetables and fruits, are associated with the lower risk of obesity and hypertension, all of which are critical risk factors for type 2 DM [48, 49]. Notably, Evans and colleagues proposed that dietary antioxidants could counteract oxidative stress, thereby enhancing insulin sensitivity and improving insulin secretion [50]. Fifth, adherence to the DASH diet’s beneficial effect on DM risk may stem from the consumption of low-fat dairy products. Prori studies demonstrated that higher dairy intake, particularly low-fat dairy, were correlated with a reduced risk of type 2 DM in men [51]. Lastly, the DASH diet, characterized by its emphasis on minimizing red and processed meats consumption, has been associated with DM risk. Shu et al. reported that high consumption of red meat was associated with an increased risk of type 2 DM [40]. As far as we know, red and processed meats are also rich sources of dietary iron. Epidemiological studies have shown that excessive iron stores in the body can promote insulin resistance, thereby increasing the risk of type 2 DM [52]. Additionally, processed meats often contain high levels of nitrates, nitrites, and nitrosamines, which are suspected to increase the risk of type 2 DM [40]. In summary, the aforementioned these mechanisms support the beneficial association between high adherence to the DASH diet and reduced risk of DM.

While a significant inverse relationship existed between adherence to the DASH diet and DM, our study also found a high heterogeneity (I^2^ = 89.1%; *P* < 0.001). To address this, we performed subgroup analyses based on study design(cohort vs case-control studies), country (Western vs Asian countries), age groups (>50 y vs <50 y), and comparison (Q5 vs.Q1/Q4 vs.Q1/T3 vs.T1). The results suggested that significant heterogeneity might be mainly due to the differences in age and country. Specifically, when the results were stratified by age and country, the heterogeneity decreased from 89.1% to 64.6%, and 55.0%, respectively. Although significant heterogeneity cannot be fully explored, there are several possible explanations for the high heterogeneity. First, Considering the variations in DASH diet between Eastern and Western populations, it is important to note that while RRs/HRs/ORs were calculated using the highest category of DASH diet (with the lowest category as a reference), the definition of DASH diet may exhibit slight differences across different studies. These variations contribute to the observed significant heterogeneity. Second, the results were pooled from different populations with different dietary habits, which might result in significant heterogeneity. Additionally, fourteen of included studies used FFQs to collect dietary data. Thus, recall bias about dietary intakes is inevitable. Third, despite adjustments for potential confounders have been included in all the included studies, a certain degree of residual or unmeasured confounding factors may exist. Ultimately, substantial heterogeneity remained in the subgroup analyses, indicating the existence of unmeasured confounding factors.

### Strengths and limitations

This systematic review and meta-analysis has several strengths and limitations. First, to our knowledge, this is the first comprehensive systematic review and dose-repsonse meta-analysis to clarify the association between adherence to the DASH diet and risk of DM. Our findings add the existing evidence regarding the beneficial effect of high adherence to the DASH diet on DM. Second, the rigorous selection of articles was carried out according to the pre-determined inclusion and exclusion criteria. Third, there were no obvious signs of publication bias in the funnel plot, and the Egger’s and Begg’s tests for publication bias were non-significant. Fourth, the quality assessment showed that the majority of studies included in the present meta-analysis were of high quality. Fifth, subgroup and sensitivity analyses were used to further explore the potential sources of heterogeneity, thereby improving the accuracy of the study results. Finally, the reported ORs/RRs /HRs were multivariate and all included studies had adjusted for some potential confounders, including age, physical activity, and total energy intake, which can affect the relationship between adherence to the DASH diet and DM risk. Despite these strengths, several limitations should be taken into account when interpreting our findings. First, in our analyses, two of the included studies used a case-control design, which is more susceptible to recall and selection bias than a cohort design. Additionally, potential confounding by pre-existing and undiagnosed diseases should be acknowledged. Thus, prospective cohort studies or randomized controlled trials are needed to further confirm the exact association between DASH diet and DM risk. Second, fourteen of the included studies used a FFQ to collect dietary intake data. However, this method may have caused misclassification and resulted in the under-or overestimation of DASH diet consumption. Furthermore, dietary intake data were self-reported, which might also lead to recall and selection biases. Third, the levels of the highest and the lowest categories of DASH diet scores were inconsistent across the included studies, which might have attenuated the true association between adherence to the DASH diet and DM risk. Also, adherence to the DASH diet may have altered over the follow-up period. Studies have reported that adherence to the DASH diet can fluctuate due to changes in socioeconomic factors such as poverty, globalization, and increased access to energy-dense foods. Fourth, significant heterogeneity was found in this study. Although we performed subgroup and sensitivity analyses to explore potential sources of heterogeneity, we could not fully ascertain and explain the sources of inter-study heterogeneity. Fifth, although high adherence to the DASH diet was associated with a decreased risk of DM, the results should be interpreted with caution. Meanwhile, there was also inconsistent adjustment for potential confounders in the included studies. As a result, the data included in our analyses may suffer from varying degrees of completeness and accuracy. Finally, it’s important to note that this study had a geographical restriction. The majority of included studies were performed in the United States, where dietary intakes markedly differ from those in Asian countries. This limitation might reduce the heterogeneity of this meta-analysis. Hence, further large prospective studies and randomized controlled trials are needed to confirm our findings across different regions and populations.

## Conclusions

In conclusion, our study showed a significant inverse association between adherence to the DASH diet and the risk of DM. Our findings contribute to the available evidence supporting the protective role of adherence to the DASH diet against DM and highlight the importance of greater adherence to the DASH diet in the prevention of DM. However, future research, particularly well-designed prospective studies or randomized controlled trials, is needed to further confirm these findings across different geographic regions.

## Supplementary information

Supplementary figure 1

Supplementary figure 2

Supplementary figure 3

Supplementary tableS1

Supplementary figures Caption

## Data Availability

The datasets used and/or analyzed during the current study are available from the corresponding author on reasonable request.

## Appendix

Table 3

**Table 3:** DASH diet and DM: assessment of study quality

Studies	Selection				Comparability		Outcome			Score
	1	2	3	4	5A	5B	6	7	8	
Cohort										
Jacobs et al. [7]	*	*	*	*	*		*	*	*	********
Liese et al. [8]	*	*	*	*	*	*	*	*	*	*********
Tobias et al. [19]		*	*	*	*	*	*	*	*	********
Tobias et al. [21]		*	*		*		*	*	*	******
Glenn et al. [30]	*	*	*	*	*	*	*	*	*	*********
Chen et al. [31]	*	*	*	*	*	*	*	*	*	*********
De Koning et al. [32]		*	*	*	*		*	*	*	*******
Jacobs et al. [33]	*	*	*	*	*		*	*	*	********
Otto et al. [22]	*	*	*	*	*		*	*	*	********
Mirmiran et al. [20]	*	*	*		*		*	*	*	*******
Esfandiar et al. [34]	*	*	*		*		*	*	*	*******
Lin et al. [35]	*	*	*	*	*	*	*	*	*	*********
Drehmer et al. [36]		*	*	*	*	*	*	*	*	********
Izadi et al. [6]	*	*		*	*		*	*	*	*******
InterAct et al. [37]	*	*	*	*	*		*	*	*	********

*For case-control studies, 1 indicates cases independently validated; 2, cases are representative of population; 3, community controls; 4, controls have no history of blood pressure disease; 5A, study controls for age; 5B, study controls for additional factor(s); 6, ascertainment of exposure by blinded interview or record; 7, same method of ascertainment used for cases and controls; and 8, non response rate the same for cases and controls. For cohort studies, 1 indicates exposed cohort truly representative; 2, non exposed cohort drawn from the same community; 3, ascertainment of exposure; 4, outcome of interest not present at start; 5A, cohorts comparable on basis of age; 5B, cohorts comparable on other factor(s); 6, quality of outcome assessment; 7, follow-up long enough for outcomes to occur; and 8, complete accounting for cohorts.
