# Supplementary figures and images for: Adherence to the dietary approaches to stop hypertension diet reduces the risk of diabetes mellitus: a systematic review and dose-response meta-analysis
Source: Endocrine. 2024 May 30;86(1):85–100. doi: 10.1007/s12020-024-03882-5 (PMC11445359; doi:10.1007/s12020-024-03882-5)

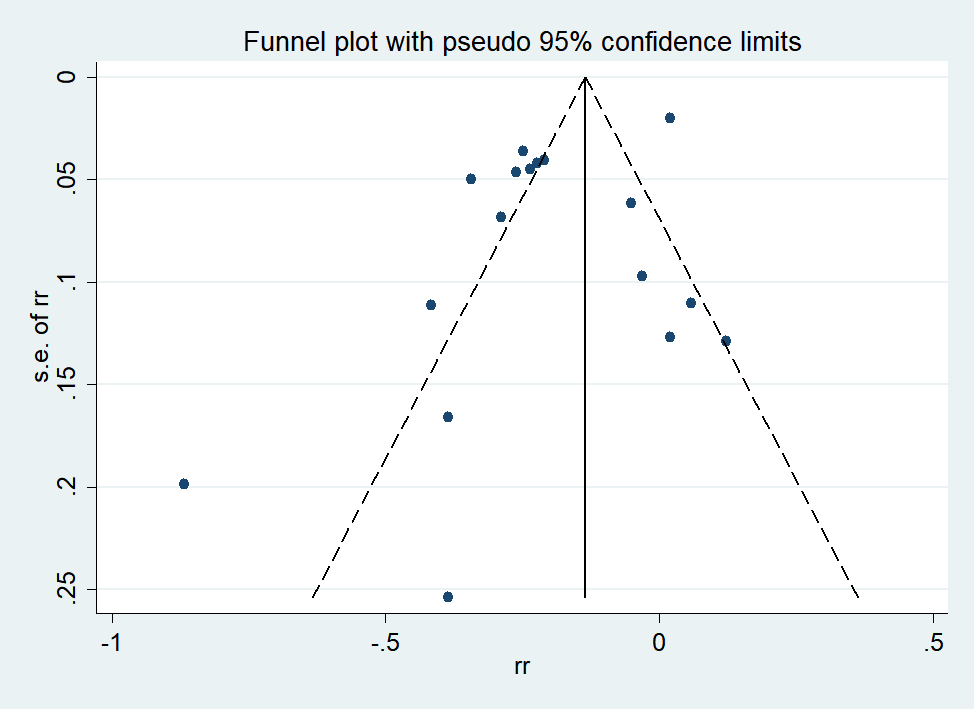

Supplement: Supplementary file 1 — Supplementary figure 1 [file 12020_2024_3882_MOESM1_ESM.tif]

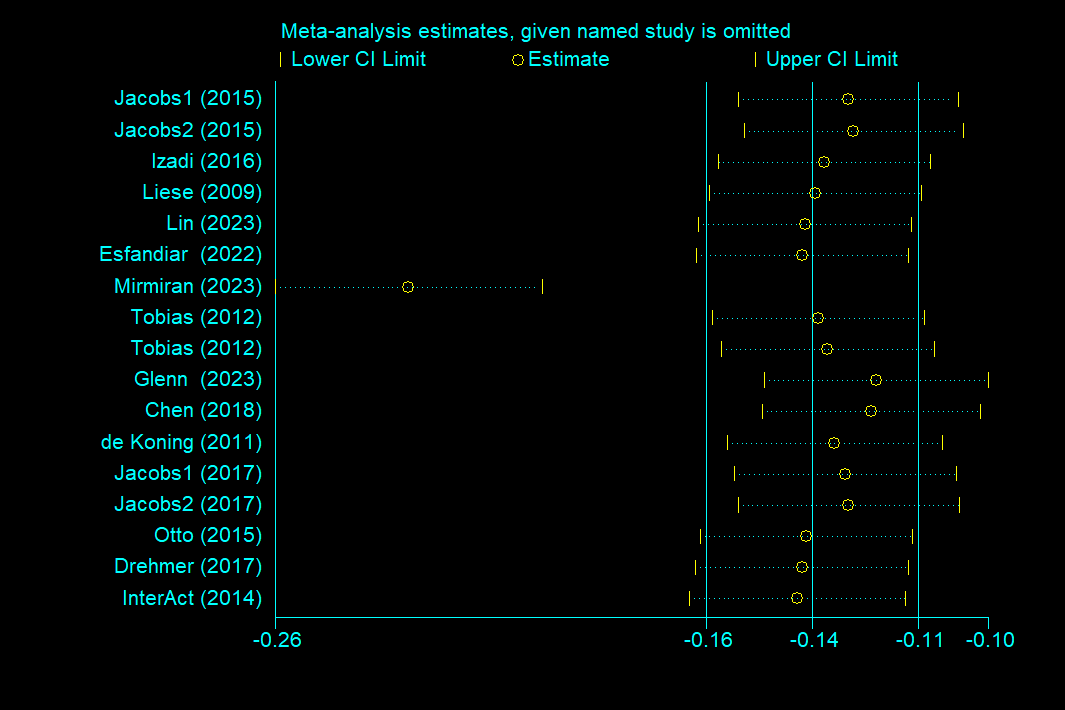

Supplement: Supplementary file 2 — Supplementary figure 2 [file 12020_2024_3882_MOESM2_ESM.tif]

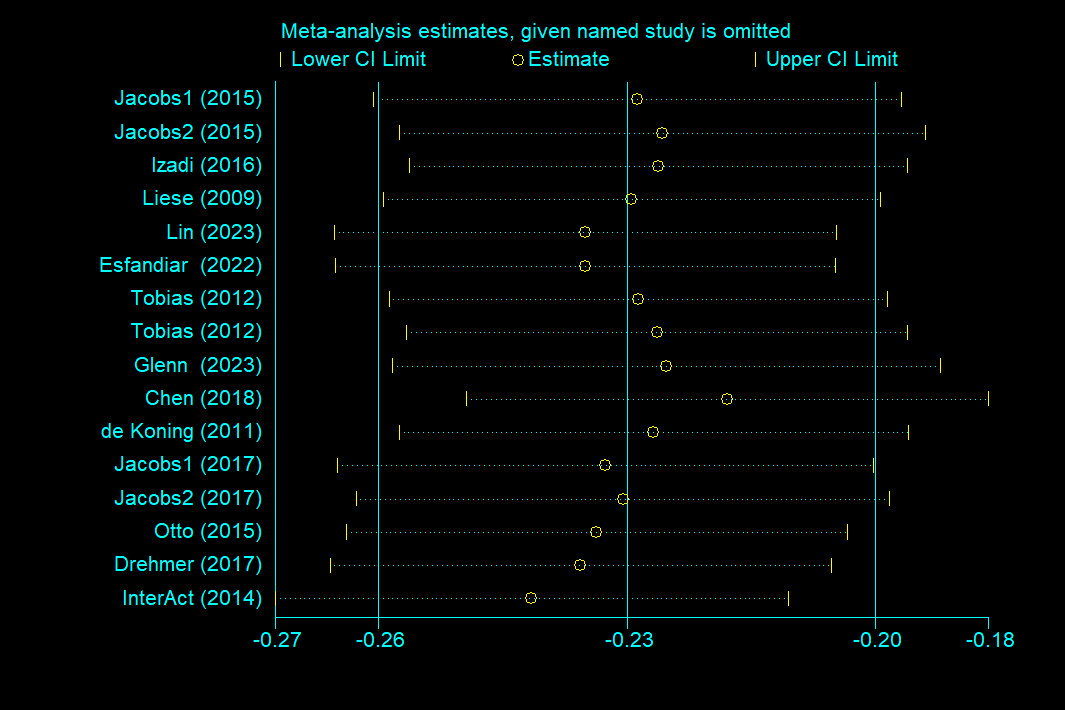

Supplement: Supplementary file 3 — Supplementary figure 3 [file 12020_2024_3882_MOESM3_ESM.tif]
