# Supplementary material for: Adherence to the dietary approaches to stop hypertension diet reduces the risk of diabetes mellitus: a systematic review and dose-response meta-analysis
Source: Endocrine. 2024 May 30;86(1):85–100. doi: 10.1007/s12020-024-03882-5 (PMC11445359; doi:10.1007/s12020-024-03882-5)
Supplement: Supplementary file 4 — Supplementary tableS1 [file 12020_2024_3882_MOESM4_ESM.doc]

Table S1. PECOS criteria for inclusion of studies

| **Population** | **Adults(aged ≥18 years)** |
| --- | --- |
| Exposure | DASH diet |
| Comparison | Highest vs. lowest categories of exposure |
| Outcomes | Diabetes mellitus |
| Study design | Observational studies(case-control, cross-sectional or cohort studies) |

PECOS, participant, exposure, comparison, outcome, and study design; DASH:Dietary Approaches To Stop Hypertension.
