# Supplementary material for: Adherence to the dietary approaches to stop hypertension diet reduces the risk of diabetes mellitus: a systematic review and dose-response meta-analysis
Source: Endocrine. 2024 May 30;86(1):85–100. doi: 10.1007/s12020-024-03882-5 (PMC11445359; doi:10.1007/s12020-024-03882-5)
Supplement: Supplementary file 5 — Supplementary figures Caption [file 12020_2024_3882_MOESM5_ESM.docx]

**Supplementary Fig.1.** The funnel plots of the association between adherence to the DASH diet and risk of DM.

**Supplementary Fig.2.** Sensitivity analysis of the association between adherence to the DASH diet and risk of DM.

**Supplementary Fig.3.** Sensitivity analysis of the association between adherence to the DASH diet and risk of DM after a cohort study was excluded
